# Supplementary material for: Searching the web builds fuller picture of arachnid trade
Source: Commun Biol. 2022 May 19;5:448. doi: 10.1038/s42003-022-03374-0 (PMC9120460; doi:10.1038/s42003-022-03374-0)
Supplement: Supplementary file 2 — Supplementary Information [file 42003_2022_3374_MOESM2_ESM.pdf]

Supplementary Materials for

Searching the web builds fuller picture of arachnid trade

Benjamin Michael Marshall, Colin Strine, Caroline S. Fukushima, Pedro Cardoso, Michael C. Orr, Alice C. Hughes*
*Corresponding author. Email: ach_conservation2@hotmail.com

Figs. S1 to S11

Supplementary Figures

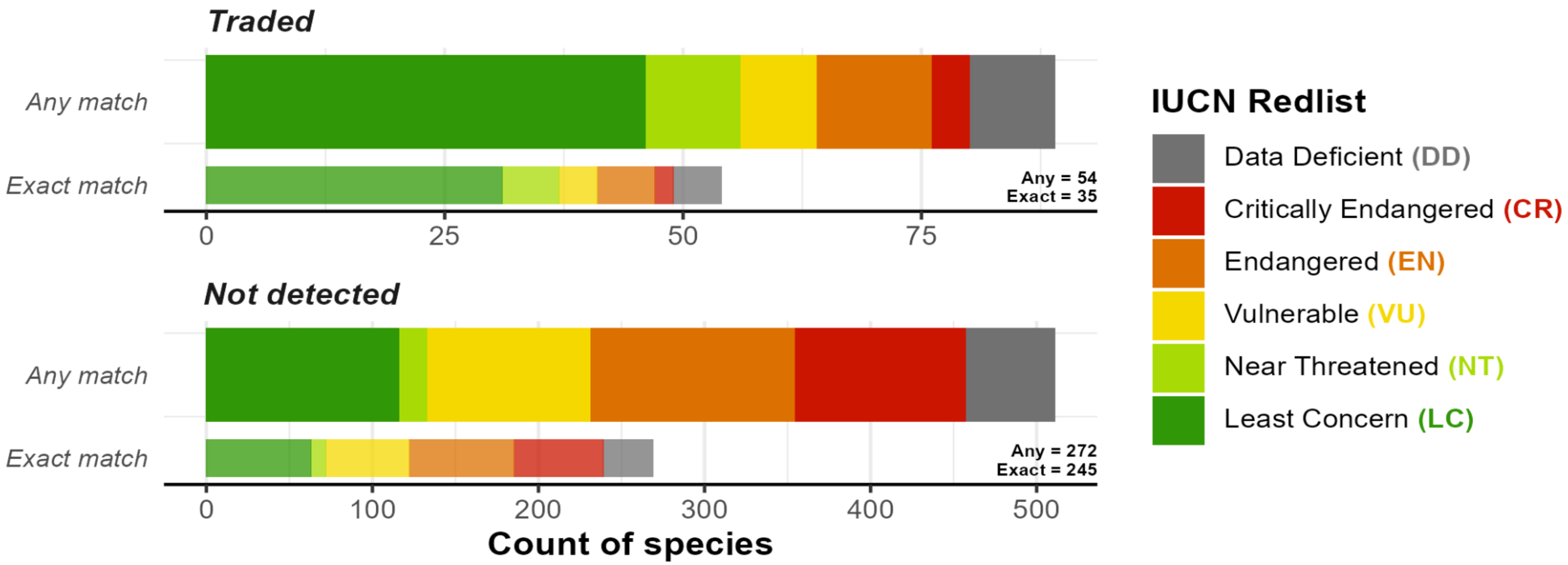

Supplementary Fig. S1 - Number of species of arachnid with an IUCN classification both detected in trade, and not in trade (not detected). Exact match signifies the species name was detected in trade, "any match" indicates that a synonym was detected in trade, but as the synonym may have been split into several taxa this can inflate the number of species potentially detected in trade. Note that most arachnids have no IUCN listing, and taxonomy has changed for some listed groups.

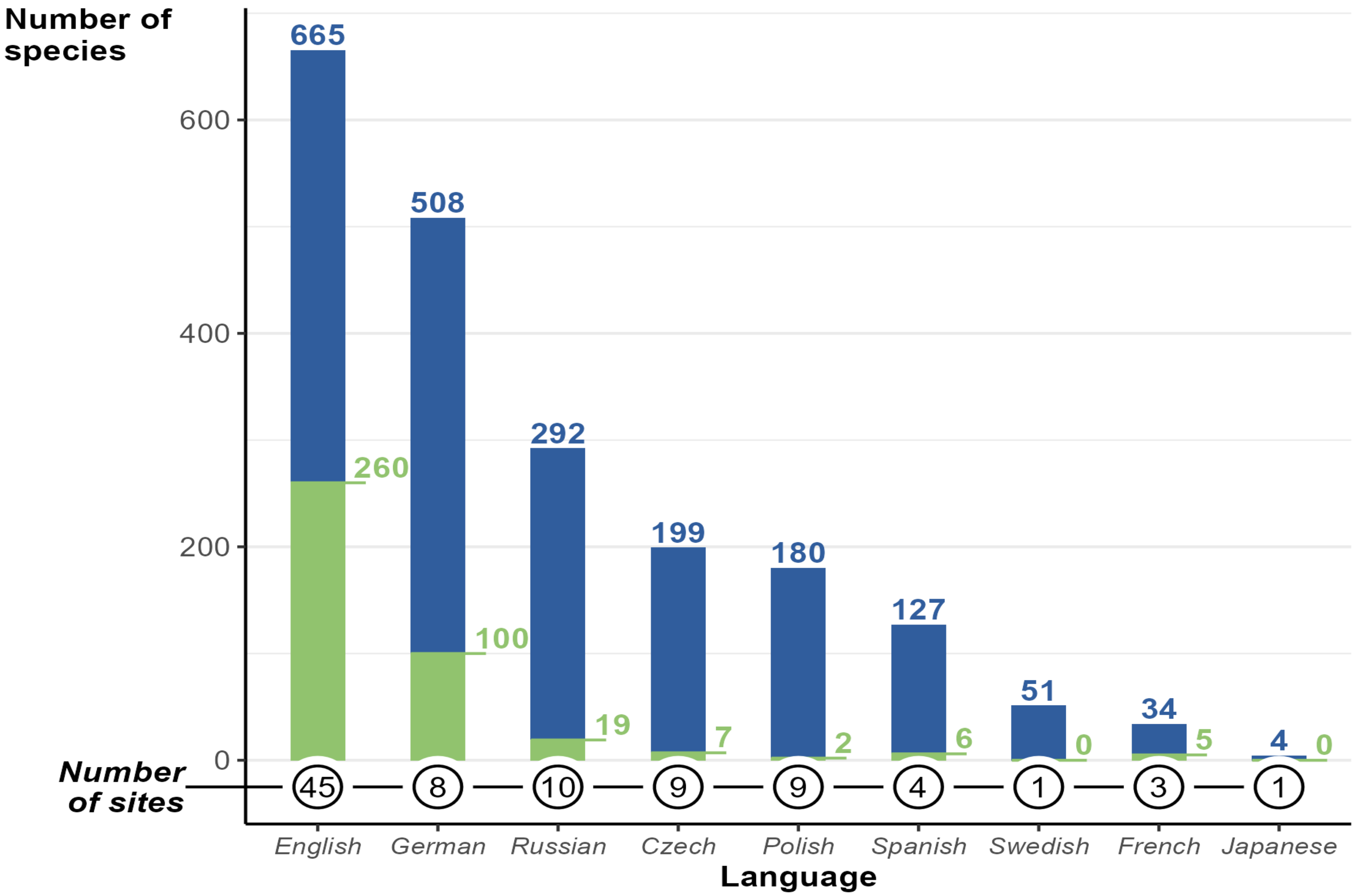

Supplementary Fig. S2 - The number of species detected online via each language search engine. Green areas and numbers show the number of species unique to that language search. The circled numbers below indicate the number of websites searched.

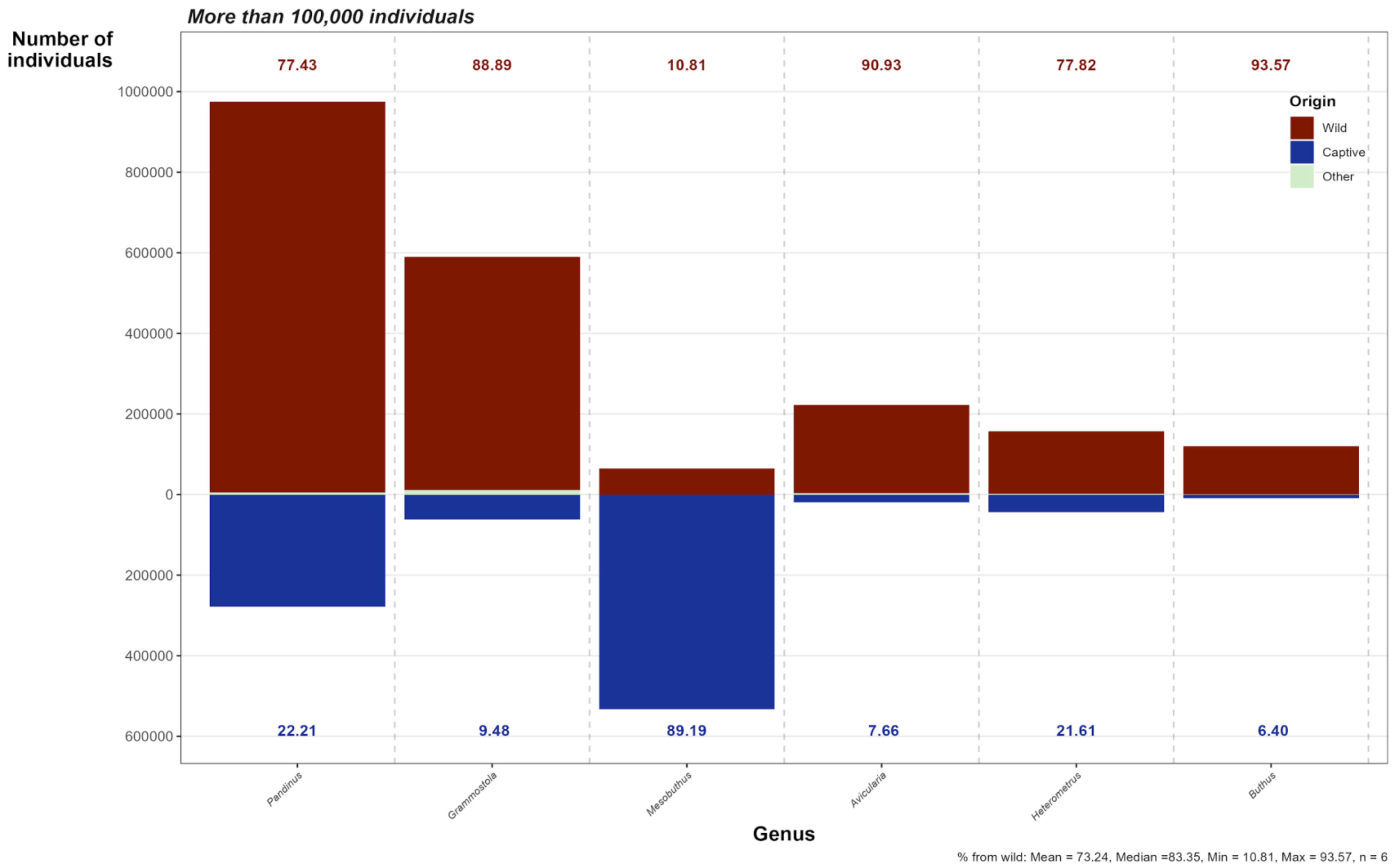

Supplementary Fig. S3 - The numbers of wild-caught versus captive originating individuals listed in LEMIS trade database, for genera with over 100,000 individuals imported.

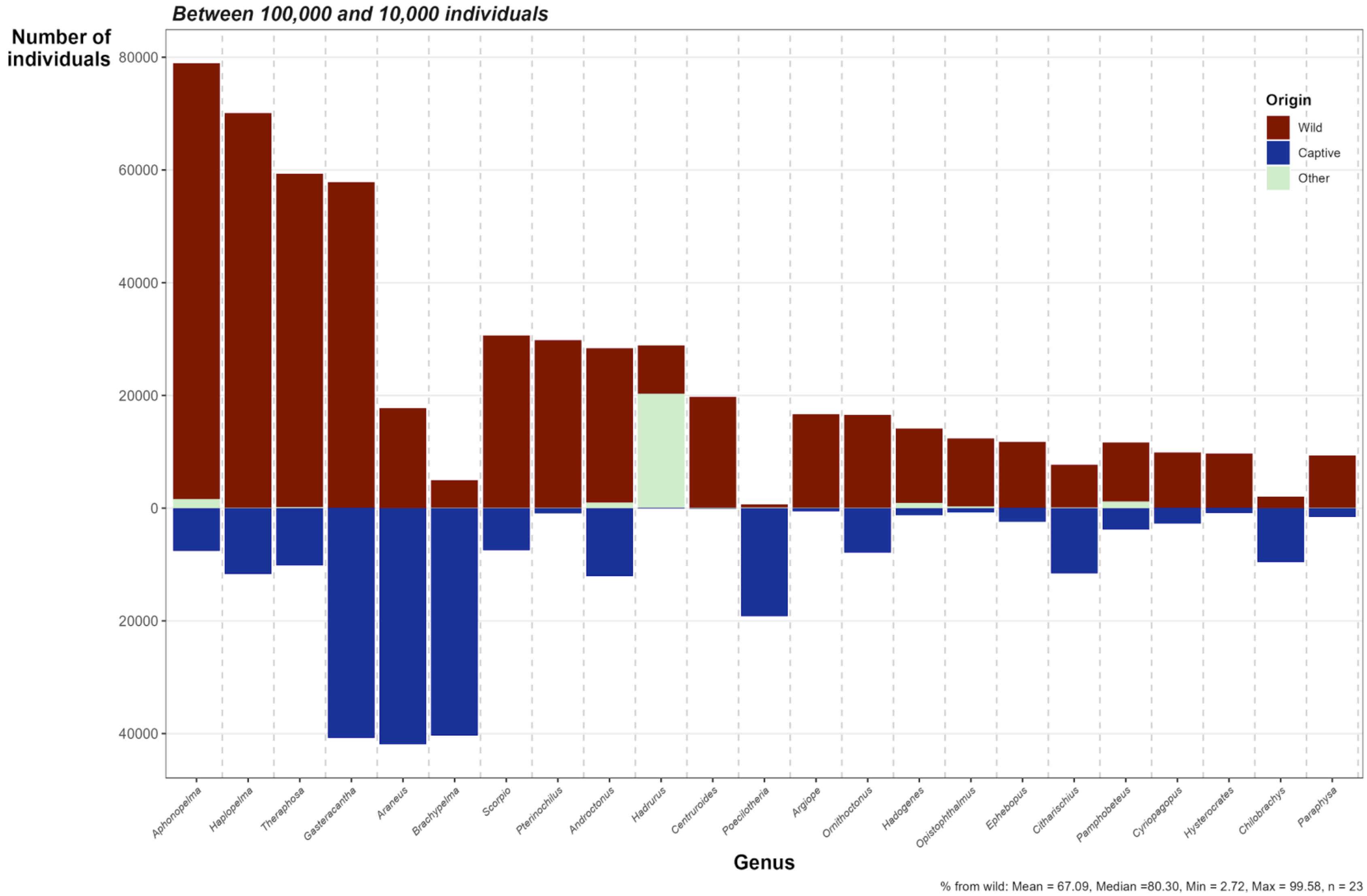

Supplementary Fig. S4 - The numbers of wild-caught versus captive originating individuals listed in LEMIS trade database, for genera with 100,000 to 10,000 individuals imported.

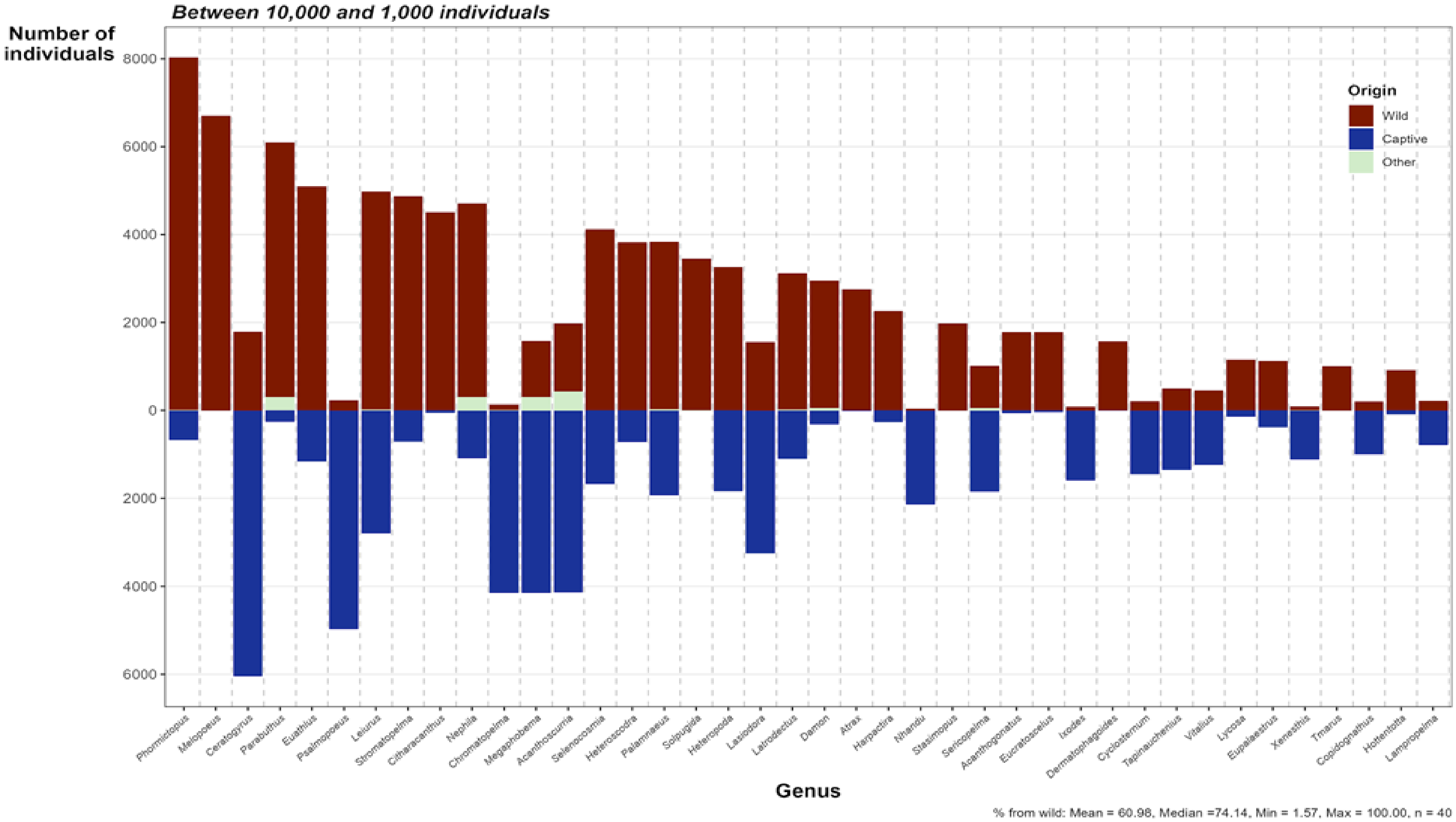

Supplementary Fig. S5 - The numbers of wild-caught versus captive originating individuals listed in LEMIS trade database, for genera with 10,000 to 1,000 individuals imported.

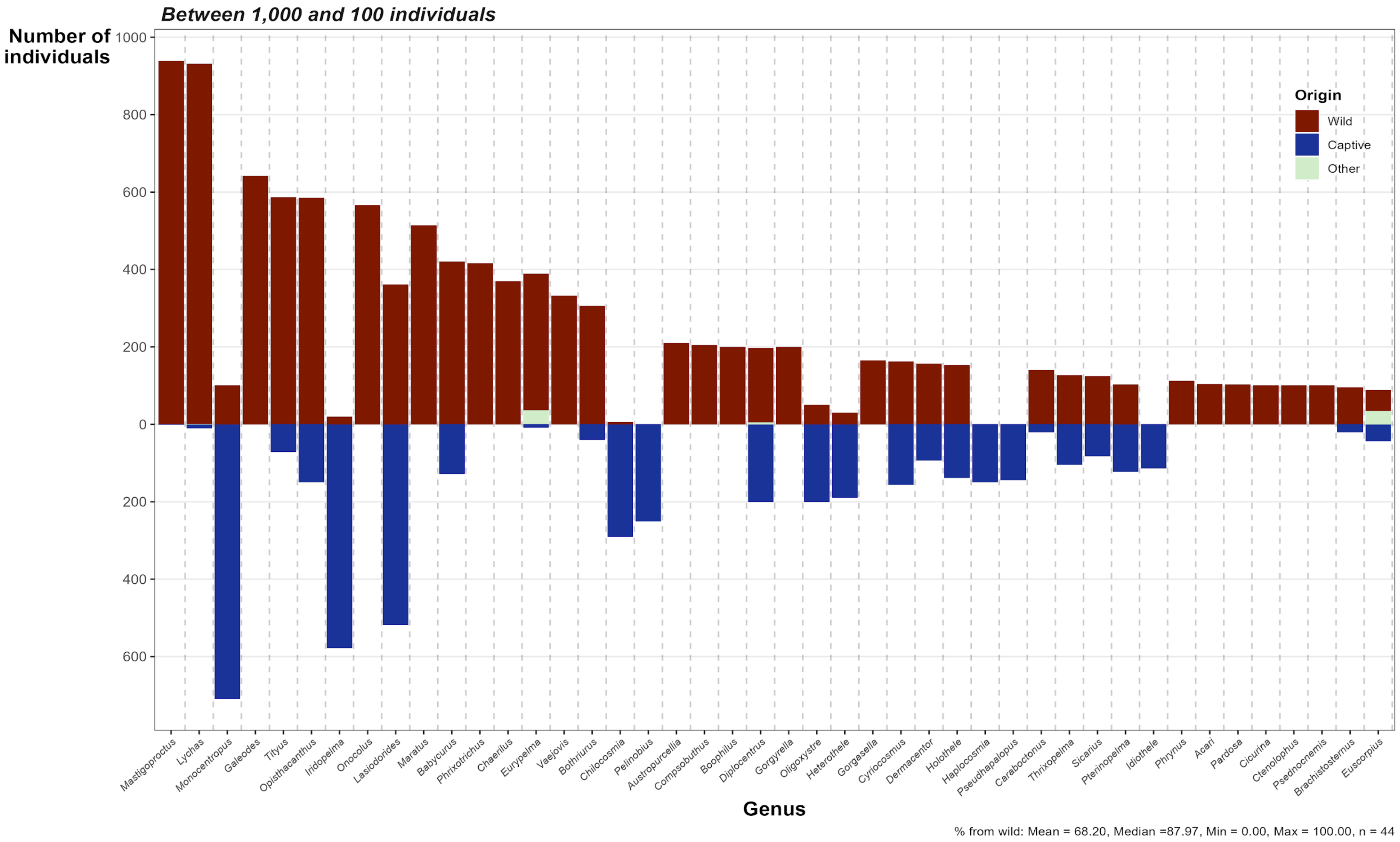

Supplementary Fig. S6 - The numbers of wild-caught versus captive originating individuals listed in LEMIS trade database, for genera with 1,000 to 100 individuals imported.

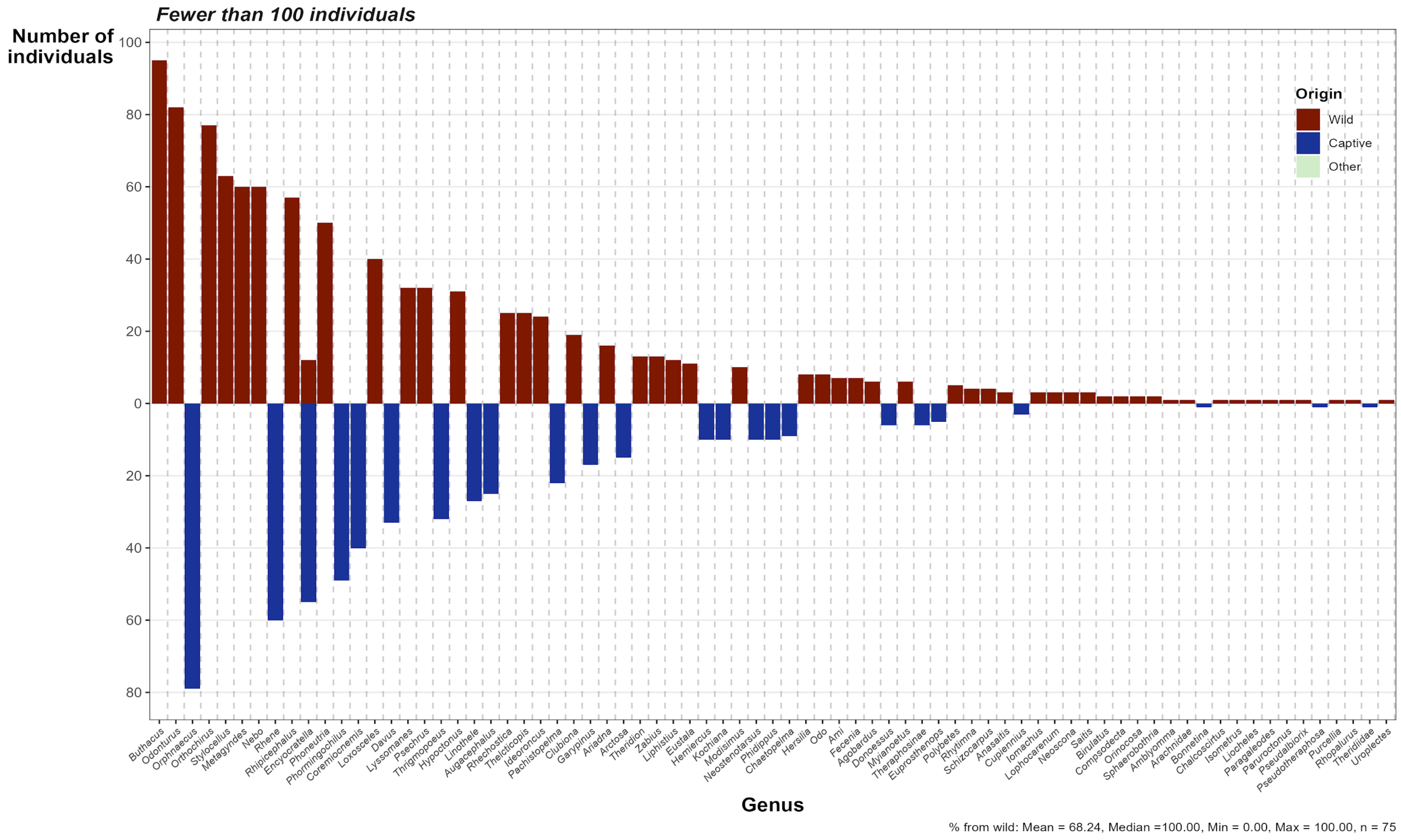

Supplementary Fig. S7 - The numbers of wild-caught versus captive originating individuals listed in LEMIS trade database, for genera with fewer than 100 individuals imported.

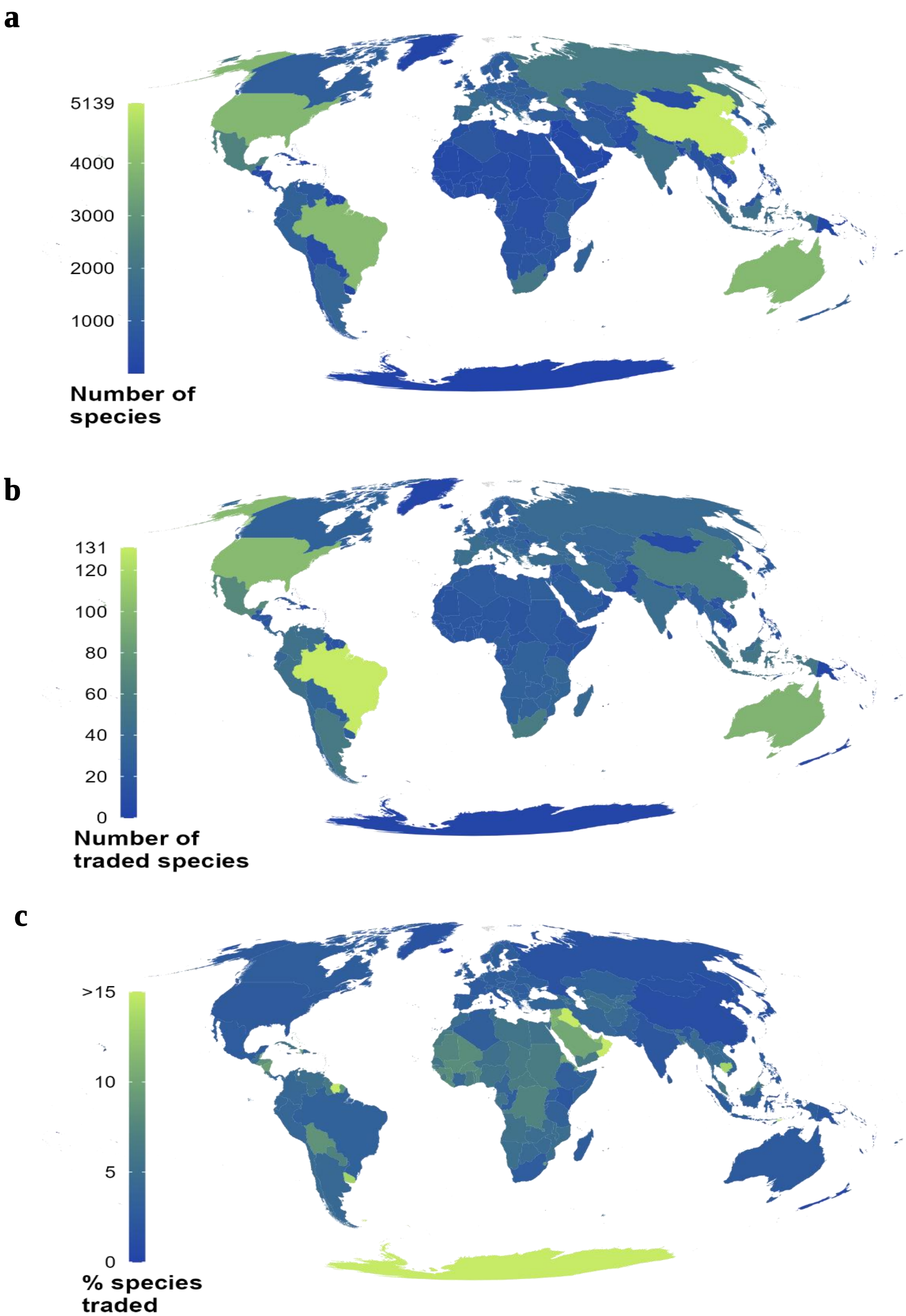

Supplementary Fig. S8 - Mapped spider species richness and traded species using WSC distribution data. a). All species richness. b). Species in trade. c). Percent species in trade.

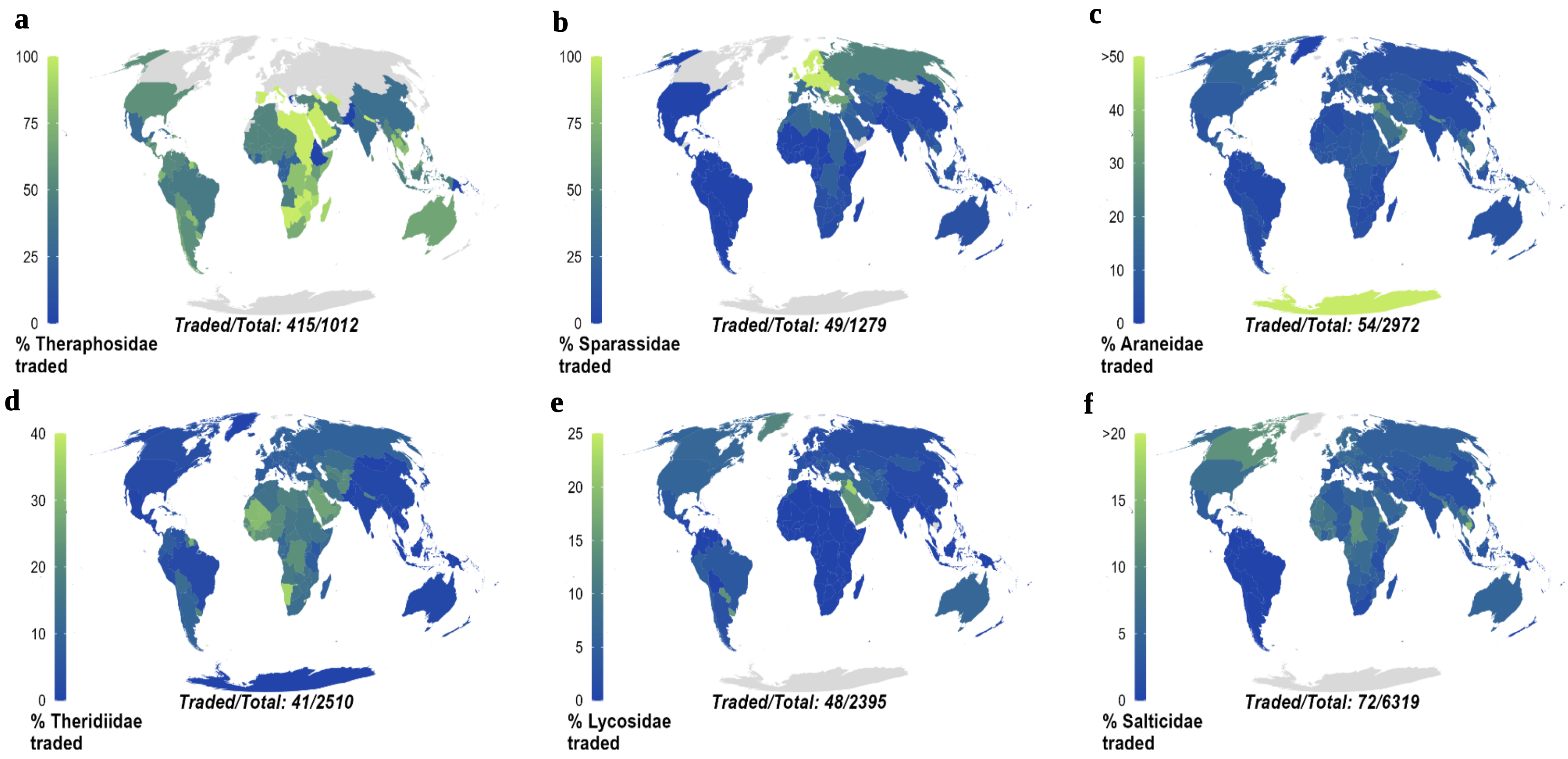

Supplementary Fig. S9 - Top six traded spider families with the percentage of species traded, using WSC distributions.

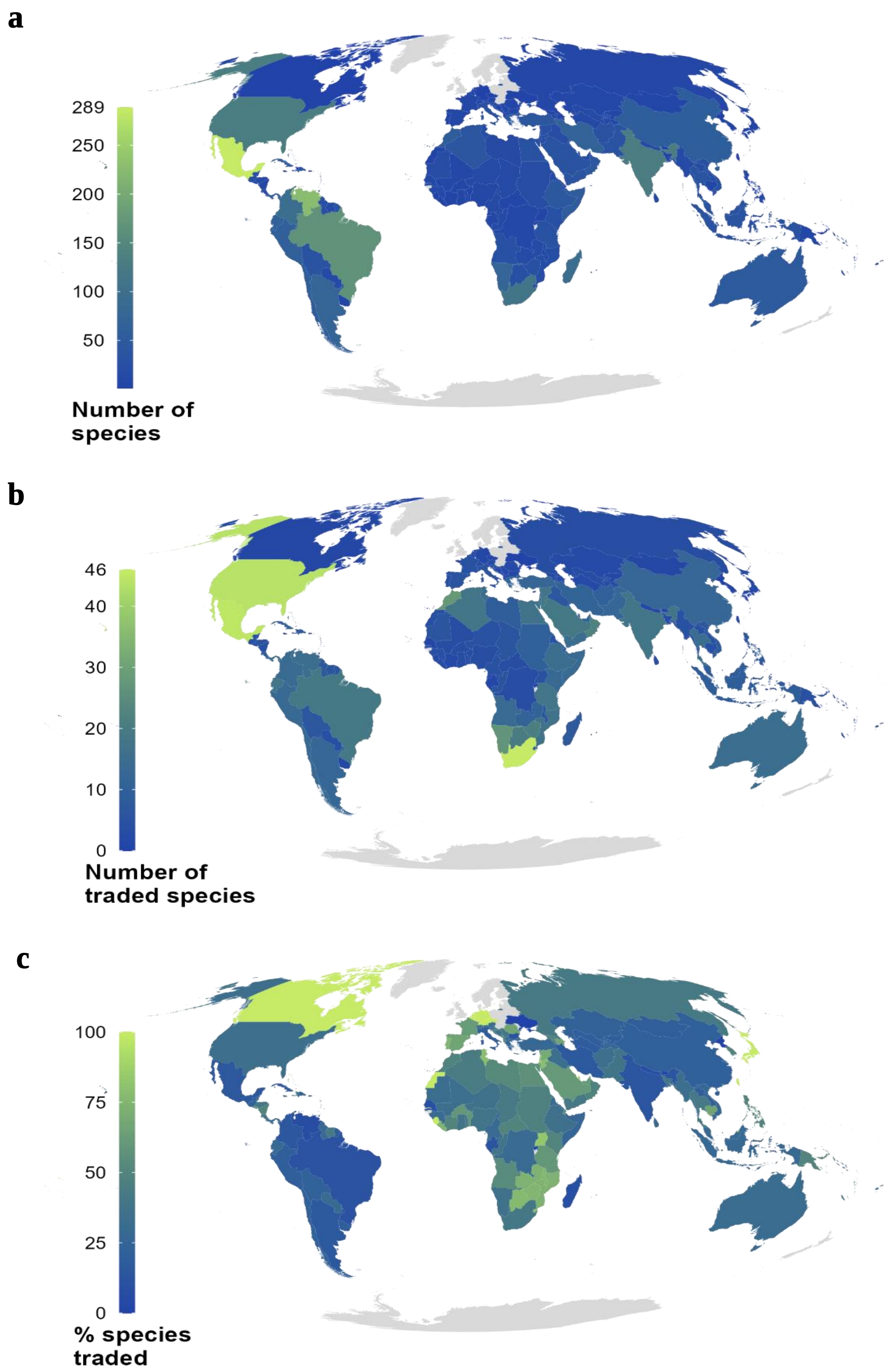

Supplementary Fig. S10 - Mapped scorpion species richness and traded species. a). All species richness. b). Species in trade. c). Percent species in trade.

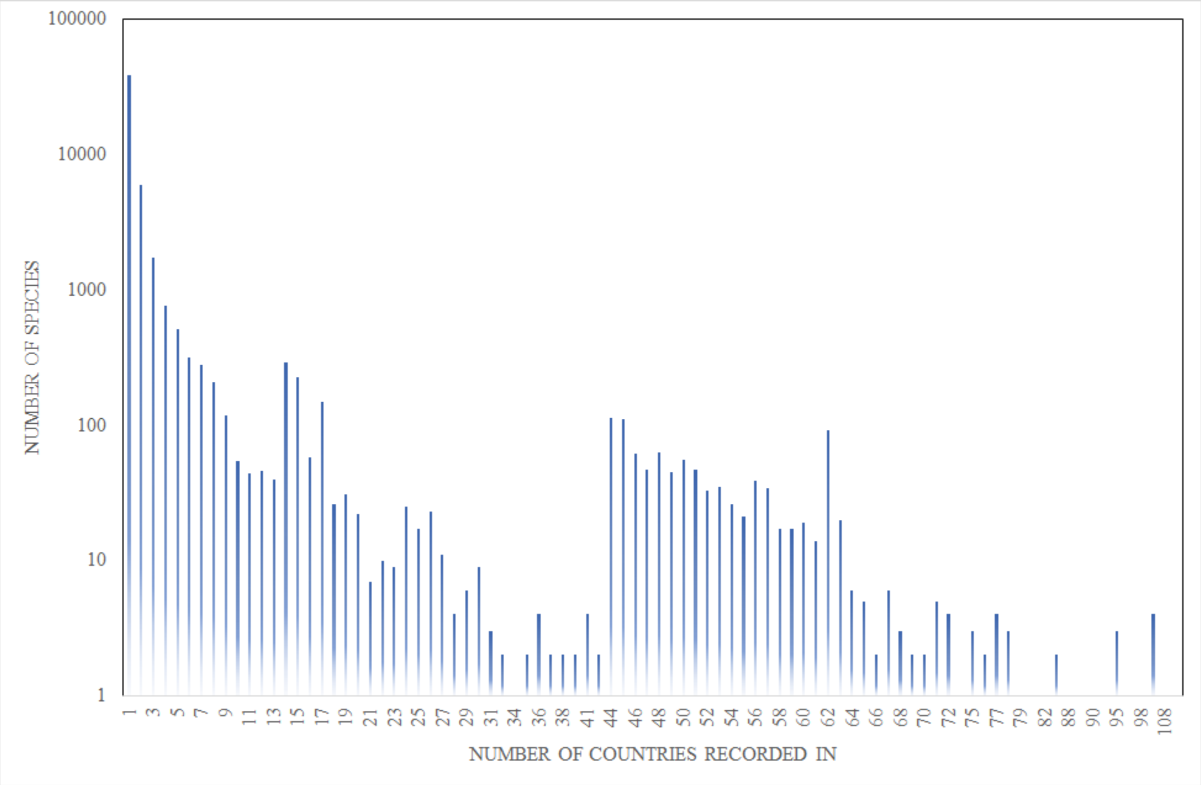

Supplementary Fig. S11. Number of countries each species are recorded in. The majority (76.4%) of species are recorded in a single country, and 94% are recorded in under five. Note log scale on the y-axis.

Supplementary References

1. Eskew, E. A. et al. United States LEMIS wildlife trade data curated by EcoHealth Alliance. Zenodo Dataset (2019) doi:10.5281/zenodo.3565869.

2. Eskew, E. A. et al. United States wildlife and wildlife product imports from 2000–2014. Sci Data 7, 22 (2020). doi:10.1038/s41597-020-0354-5

3. UNEP-WCMC (Comps.). CITES trade statistics derived from the CITES Trade Database, UNEP World Conservation Monitoring Centre, Cambridge, UK. https://trade.cites.org. [2021-09-15] (2021)

4. UNEP-WCMC (Comps.). The Checklist of CITES Species Website. CITES Secretariat, Geneva, Switzerland. Compiled by UNEP-WCMC, Cambridge, UK. Available at: http://checklist.cites.org. [2019-09-15] (2021)

5. IUCN. The IUCN Red List of Threatened Species. https://www.iucnredlist.org [2021-09-15] (2021).
