# Supplementary material for: Searching the web builds fuller picture of arachnid trade
Source: Commun Biol. 2022 May 19;5:448. doi: 10.1038/s42003-022-03374-0 (PMC9120460; doi:10.1038/s42003-022-03374-0)
Supplement: Supplementary file 3 — Description of Additional Supplementary Files (editable) [file 42003_2022_3374_MOESM3_ESM.pdf]

Description of other Supplementary Materials for this manuscript include the following:

Data S1 to S14

Code S1 to S13

Supplementary References 1 to 5

Data S1. (Separate file) - Website data: lang = language of the search engine used, ad hoc websites had language described after discovery; engine = the search engine used; page = the page on which the website appeared from the search engine; searchdate = search date in YYYY-mm-dd HH:MM:SS; link = link to the webpage, redacted to protect website identity; reviewdate = date revewied for arachnids being sold and search strategy; sells = whether the website sells arachnids (1 == sells); allow = whether the site explicitly forbids automated searching (1 == allows, NA when search method was not fully automated, e.g., single page); type = the type of the website (e.g., trade, classified ads); order = whether arachnids where organised in a particular ways; target = a refined target URL to start search; method = the search method chosen, see methods for details; refine = any refinement or filter than could constrain the scope of the website to be searched; spages = the number of pages required to cycle through to cover the entire stock (also separated by ; if multiple cycles where needed or multiple single pages could be easily collected); prelimCheck = whether the website passed initial checks for arachnid selling; notes = any details that might need special attention during searches; webID = code used for subsequent data summary.

Data S2. (Separate file) - Raw keyword searches outputs: species keywords. sp = the modern species or genus that a keyword is associated with; page = the number of the page the keyword was detected on; keyw = the exact keyword that was detected; spORgen = whether the keyword was a species binomial or just genus; termsSurrounding = the words surrounding a genus keyword detection (only applies to Data S3); webID = the website ID.

Data S3. (Separate file) – Raw keyword searches outputs: genus keywords. sp = the modern species or genus that a keyword is associated with; page = the number of the page the keyword was detected on; keyw = the exact keyword that was detected; spORgen = whether the keyword was a species binomial or just genus; termsSurrounding = the words surrounding a genus keyword detection (multiple detections separated by ;); webID = the website ID.

Data S4. (Separate file) - Raw keyword search outputs: temporal sample. sp = the modern species or genus that a keyword is associated with; page = the number of the page the keyword

was detected on; keyw = the exact keyword that was detected; spORgen = whether the keyword was a species binomial or just genus; termsSurrounding = the words surrounding a genus keyword detection (multiple detections separated by ;); webID = the website ID; timestamp.parse = the timestamp extracted from the archived web page; year = a simplified timestamp including only the year.

Data S5. (Separate file) - LEMIS data used. An arachnid filtered version of (*1*,*2*).

Data S6. (Separate file) - CITES trade database data used (*3*).

Data S7. (Separate file) - CITES appendices data used (*4*).

Data S8. (Separate file) - IUCN Redlist data used (*5*).

Data S9. (Separate file) - Compiled final dataset, with data deriving from WSC, Scorpion files, ITIS, WAM and the data collection process. speciesId = a numeric code, one per species; clade = the clade the species belongs to; family = the family the species belongs to; genus = the genus of the species; species = the species epithet; author = the species authority name; year = the species authority year; parentheses = whether parentheses are needed with the authority; distribution = WSC original distribution descriptions; invalid = whether the species is considered valid; source = the species source, either World Spider Catalogue, Scorpion files, ITIS or WAM; accName = the species binomial being used as our accepted name; allNames = the accepted species binomial and all synonyms; allGenera = the accepted genus, and all other genera the species has belonged to at one point; onlineTradeSnap = whether the species was detected via a match to the accName in the snapshot data; onlineTradeSnap_Any = whether the species was detected via any synonym in the snapshot data; onlineTradeSnap_genus = whether the genus was detected via a match to the genus in the snapshot data; onlineTradeSnap_genusAny = whether the genus was detected via any synonym in the snapshot data; onlineTradeTemp = whether the species was detected via a match to the accName in the temporal data; onlineTradeTemp_Any = whether the species was detected via any synonym in the temporal data; onlineTradeTemp_genus = whether the genus was detected via a match to the genus in the temporal data; onlineTradeTemp_genusAny = whether the genus was detected via any synonym in the temporal data; onlineTradeEither = whether the species was detected via a match to the accName in the temporal data or snapshot data; onlineTradeEither_Any = whether the species was detected via any synonym in the temporal data or snapshot data; LEMIStrade = whether the species was detected via a match to the accName in the LEMIS data; LEMIStrade_Any = whether the species was detected via any synonym in the LEMIS data; LEMIStrade_genus = whether the genus was detected via any synonym in the LEMIS data; LEMIStrade_genusAny = whether the genus was detected via any synonym in the LEMIS data; CITEStrade = whether the species was detected via a match to the accName in the CITES trade database data; CITEStrade_Any = whether the species was detected via any synonym in the CITES trade database data; CITEStrade_genus = whether the genus was detected via any synonym in the CITES trade database data; CITEStrade_genusAny = whether the genus was detected via any synonym in the CITES trade database data; CITESapp = the CITES appendix the species is listed under using an exact match to the accName; CITESapp_Any = the CITES appendix the species is listed under using any match to any of the

species’ synonyms; redlist = the IUCN Redlist category the species is listed under using an exact match to the accName; redlist_Any = the IUCN Redlist category the species is listed under using any match to any of the species’ synonyms; extactMatchTraded = the species is detected in any of the trade sources via a match to the accName; anyMatchTraded = the species is detected in any of the trade sources via a match to any species’ synonym.

Data S10. (Separate file) - Forum listings of “What species are you currently keeping” from an online fora posted between 9th September 2021 and 9th October 2021, to provide an idea of online discussions. Each user with a separate list is provided in a separate tab. Morph_collector is the same as poster1, but the potential cryptic species or morphs are noted separately to make them clearer.

Data S11. (Separate file) – Shapefiles from thematicmapping used in mapping summaries.

Data S12. (Separate file) – Distribution information for spiders. Only two columns used in summaries: accName = the accepted name used throughout summaries; NAME = the country name the spider occurs in.

Data S13. (Separate file) - Distribution information for scorpions. species = the accepted name used throughout summaries; NAME = the country name the scorpions occurs in.

Data S14. (Separate file) Trends in searches of certain search words relating to arachnids

Code S1. (Separate file) - Search URL Extract.R

Code S2. (Separate file) - Retrieve web data.R

Code S3. (Separate file) - Temporal Classified Ads.R

Code S4. (Separate file) - Keyword Generation.R

Code S5. (Separate file) - Keyword Search.R

Code S6. (Separate file) - LEMIS filter and summary.R

Code S7. (Separate file) - Compiling results.R

Code S8. (Separate file) - Summary Figures.R

Code S9. (Separate file) - Temporal Figures.R

Code S10. (Separate file) - New description figure.R

Code S11. (Separate file) - Term exploration.R

Code S12. (Separate file) - LEMIS summary and mapping.R
